# Explainable Deep Ensemble Meta-Learning Framework for Brain Tumor Classification Using MRI Images

**DOI:** 10.3390/cancers17172853

**Published:** 2025-08-30

**Authors:** Shawon Chakrabarty Kakon, Zawad Al Sazid, Ismat Ara Begum, Md Abdus Samad, A. S. M. Sanwar Hosen

**Affiliations:** 1 Department of Artificial Intelligence and Big Data, Woosong University, Daejeon 34606, Republic of Korea; chakrabartyshawon@gmail.com (S.C.K.); zawadsazid44@gmail.com (Z.A.S.); 2 Department of Biomedical Sciences and Institute for Medical Science, Jeonbuk National University Medical School, Jeonju 54907, Republic of Korea; ismatara1986@gmail.com; 3 Department of Information and Communication Engineering, Yeungnam University, Gyeongsan 38541, Republic of Korea

**Keywords:** brain tumor detection, MRI images, deep learning, explainable artificial intelligence

## Abstract

**Background:** Brain tumors can severely impair neurological function, leading to symptoms such as headaches, memory loss, motor coordination deficits, and visual disturbances. In severe cases, they may cause permanent cognitive damage or become life-threatening without early detection. **Methods:** To address this, we propose an interpretable deep ensemble model for tumor detection in Magnetic Resonance Imaging (MRI) by integrating pre-trained Convolutional Neural Networks—EfficientNetB7, InceptionV3, and Xception—using a soft voting ensemble to improve classification accuracy. The framework is further enhanced with a Light Gradient Boosting Machine as a meta-learner to increase prediction accuracy and robustness within a stacking architecture. Hyperparameter tuning is conducted using Optuna, and overfitting is mitigated through batch normalization, L2 weight decay, dropout, early stopping, and extensive data augmentation. **Results:** These regularization strategies significantly enhance the model’s generalization ability within the BR35H dataset. The framework achieves a classification accuracy of 99.83 on the MRI dataset of 3060 images. **Conclusions:** To improve interpretability and build clinical trust, Explainable Artificial Intelligence methods Grad-CAM++, LIME, and SHAP are employed to visualize the factors influencing model predictions, effectively highlighting tumor regions within MRI scans. This establishes a strong foundation for further advancements in radiology decision support systems.

## Simple Summary

Detecting brain tumors early on Magnetic Resonance Imaging (MRI) scans is challenging. We developed an AI model that analyzes MRI images to determine the presence of tumors. By combining multiple pre-trained Deep Learning models, the system reduces errors and provides a final decision with high accuracy on standard public datasets. It also highlights the regions that influenced its prediction, allowing clinicians to interpret and verify results. This transparent and reproducible framework aims to serve as a foundation for the research community, supporting the development of reliable tools for screening, review, and follow-up while reducing diagnostic workload and prioritizing patient safety.

## 1. Introduction

Brain tumor detection in medical imaging plays a critical role in modern healthcare, influencing early diagnosis, treatment planning, and ultimately patient outcomes. Accurate brain tumor detection within complex image datasets is vital for improving survival rates and minimizing treatment-related complications. However, this task remains challenging due to considerable variability in tumor size, shape, and intensity, as well as the presence of noise and artifacts in medical images. Traditional diagnostic methods, which often rely on manual interpretation by radiologists, are time-consuming and inherently prone to variability and human error [1]. These challenges highlight the urgent need for automated, scalable, and reliable solutions in clinical practice. Early brain tumor detection has been associated with a five-year survival rate increase from 15% to nearly 75% in certain tumor types [2]. Inter-observer variability among radiologists can reach up to 30% in brain MRI interpretation, posing a risk to consistent diagnosis and treatment planning [3].

Several recent studies have exclusively utilized the BR35H binary dataset (1500 tumor and 1500 non-tumor Magnetic Resonance Imaging (MRI) scans) to advance brain tumor classification techniques. Saeed et al. [4] developed a dual Deep Convolutional Neural Network (DCNN) (DenseNet121 + InceptionV3) and achieved 99% accuracy, 99% precision, and 98% recall using the BR35H dataset. Similarly, Amran et al. [5] proposed a hybrid architecture combining GoogLeNet with a custom CNN, attaining 99.51% accuracy, 99% precision, 98.90% recall, and 98.50% F1-score on the same dataset. In another study, Muskan et al. [6] applied EfficientNet-B2 exclusively to BR35H, reporting a validation accuracy of up to 99.83% on the binary classification task. Furthermore, Khan and Auvee [7] conducted a comparative analysis of lightweight CNN models such as ResNet-18, VGG-16, and a custom CNN, achieving a maximum accuracy of 98.67% on BR35H. While these works demonstrate exceptional performance using BR35H, most are limited to single or dual-model pipelines and do not incorporate advanced ensemble or interpretability frameworks.

The proposed model integrates extensive data augmentation and advanced regularization techniques, including batch normalization and L2 (Ridge) weight decay, to enhance generalization. Hyperparameter optimization is performed using Optuna [8], ensuring a balanced trade-off between model complexity and computational efficiency. Explainable Artificial Intelligence (XAI) methods are incorporated to strengthen clinical trust: Gradient-Weighted Class Activation Mapping++ (Grad-CAM++) [9] provides spatial localization by highlighting MRI regions most influential to predictions. Local Interpretable Model-Agnostic Explanations (LIME) and Shapley Additive Explanations (SHAP) provide complementary interpretability: LIME approximates the model’s behavior around a single case (local), while SHAP quantifies each feature’s contribution to the overall prediction (global) [10]. These complementary techniques ensure both local and global transparency, which is critical for clinical adoption. The classification backbone consists of pre-trained CNN architectures—EfficientNetB7 [11], InceptionV3, and Xception [12]—whose outputs are combined via a Light Gradient Boosting Machine (LightGBM) meta-learner [13] to maximize predictive performance and robustness. By uniting high-performing CNN-based Deep Learning (DL), gradient boosting meta-learning, and multi-faceted interpretability, this framework delivers a scalable, transparent, and clinically applicable solution for automated brain tumor detection, establishing a strong benchmark for future AI-assisted medical diagnostic systems. The key contributions of this work are summarized as follows:

- Ensembled EfficientNetB7, Inception, and Xception models using soft-voting strategies to enhance robustness and address classification challenges.
- Combined CNN outputs with a LightGBM meta-learner, reducing overfitting and improving classification reliability across varying tumor appearances.
- Applied extensive data augmentation, class balancing, batch normalization, and L2 weight decay to reduce overfitting and address class imbalance.
- Achieved high accuracy on a separate test set, with near-perfect precision, recall, and F1-score. The LightGBM meta-learner was tuned and evaluated via five-fold stratified cross-validation on the training and validation data using an out-of-fold stacking strategy. Cross-validation results are reported separately from independent test performance.
- Incorporated Grad-CAM++, LIME, and SHAP to generate visual explanations.

The rest of the paper is structured as follows. Section 2 reviews state-of-the-art approaches and recent advancements in brain tumor detection using medical imaging and DL techniques. Section 3 describes the proposed framework, including its architecture, preprocessing pipeline, and integration of XAI methods. Section 4 presents the experimental results along with an in-depth analysis of the model’s performance. Section 5 provides a critical discussion of the proposed model. Finally, Section 6 concludes the work and outlines potential directions for future research.

## 2. Related Work

Advancement of machine learning (ML) and DL has enhanced the accuracy and efficiency of brain tumor diagnosis [14]. While many studies have proposed promising diagnostic models, most of them suffer from critical limitations, including higher accuracy, limited interpretability, and scalability for clinical deployment. This section reviews recent and relevant research efforts, highlighting their methodological approaches, strengths, and limitations in comparison to the proposed model.

Aamir et al. [15] proposed an optimized CNN architecture to enhance generalization performance of the tumor classification model. The authors have used advanced hyperparameter tuning and trained their model using three popularly used MRI datasets: BRATS, Figshare, and BR35H [16]. The optimized CNN achieved an average classification accuracy of 97%, which the authors attributed to several key architectural and methodological choices. The number of layers and neurons per layer were carefully selected (the use of Rectified Linear Unit (ReLU) activation functions to capture complex nonlinear patterns within MRI images are some of those), along with application of effective image preprocessing techniques such as normalization and augmentation. To address overfitting and enhance model robustness across diverse datasets, regularization strategies, particularly dropout, were used in the network design.

Dewage et al. [17] introduced a custom CNN architecture designed specifically for brain tumor classification using MRI images. The model was trained on a Kaggle-sourced dataset comprising four classes: Glioma, Meningioma, Pituitary Tumors, and Non-Tumors. To address the issue of class imbalance, the authors applied advanced data augmentation techniques including rotation, flipping, scaling, and incorporated class weighting during training. Their proposed CNN achieved a classification accuracy of 94.51%, outperforming other models such as ResNetV2, DenseNet201, and VGG16 in terms of accuracy, F1-score, precision, and recall. To prevent overfitting, the model utilized dropout regularization, early stopping, and L2 regularization. A key contribution of the study was its strong focus on interpretability; the authors implemented gradient-based attribution methods, including integrated gradients, guided backpropagation, and saliency maps, to provide visual explanations of the model’s predictions. The study emphasized that in neuro-oncology, model transparency is essential for clinical acceptance and decision-making support, making interpretability a central pillar alongside accuracy.

Al-Zoghby et al. [18] proposed a hybrid DL model that integrates a pre-trained VGG16 network with a custom-designed CNN in dual-stream architecture for brain tumor classification. This model was trained and validated on an open-source Figshare dataset [19] comprising 3064 MRI images across three tumor types: Glioma, Meningioma, and Pituitary. The VGG16 stream extracts rich hierarchical features, while the custom CNN branch is tailored for task-specific learning, enabling the model to capture both low-level and high-level tumor characteristics. By merging the outputs of both branches through a concatenation layer followed by dense layers, the architecture achieved a test accuracy of 99%, demonstrating high generalization capability and robustness in multiclass tumor classification.

Asiri et al. [20] proposed a hybrid DL framework that combines a fine-tuned ResNet50 for brain tumor classification with a U-Net architecture for tumor segmentation. The model was trained and evaluated on two publicly available datasets: TCGA-LGG and TCIA [21]. The classification component achieved an accuracy of 94%, while the segmentation module reported a Dice Similarity Coefficient (DSC) of 0.95 and an Intersection over Union (IoU) of 0.91, indicating strong agreement with expert-annotated ground truth. The U-Net architecture, known for its skip connections, effectively preserved spatial and contextual features during segmentation. Meanwhile, ResNet50’s residual blocks enabled deep feature extraction, contributing to accurate tumor classification. This dual-stream framework addresses both tumor localization and diagnosis, showcasing the advantages of combining transfer learning and specialized architectures for multi-task learning. Even when trained on relatively limited data, their approach demonstrated robust generalization and clinical relevance, establishing a strong benchmark for integrated brain tumor analysis.

Chinga et al. [22] conducted a comparative study of CNN architectures for brain tumor classification using MRI scans, including AlexNet, VGG16, LeNet, LeNet5-like, and ZFN Net. Among them, AlexNet (version 1) achieved an average training accuracy of 99.84% and a validation accuracy of 95.19%, while the ZFN Net (version 1) slightly outperformed it with a validation accuracy of 96.32%. The study placed strong emphasis on diagnostic interpretability by integrating Grad-CAM, a widely used visualization technique that highlights image regions most influential in the model’s decision-making. This approach supports clinical decision-making by enhancing transparency and trust in AI-based diagnostics. The findings also underline the importance of architectural efficiency and context-aware training, especially in scenarios with limited annotated data. Moreover, the study emphasized the critical role of transfer learning in developing accurate, stable, and data-efficient DL models for brain tumor classification.

Ahamed and Sadia [23] investigated the impact of transfer learning on brain tumor classification using CNNs. Their study evaluated EfficientNet-B5 and ResNet50, both pre-trained on ImageNet, for two tasks: binary classification (tumor vs. non-tumor) and multi-class classification (Glioma, Meningioma, and Pituitary). MRI data were sourced from two datasets, Figshare (2015) and SARTAJ [24,25]. Among the models assessed, EfficientNet-B5 achieved the best performance, reaching 99.75% accuracy for binary classification and 98.61% for multi-class classification, outperforming ResNet50. The authors attributed this strong performance to EfficientNet’s compound scaling strategy and optimized convolutional blocks, which enhance learning efficiency, particularly in low-data settings. The study emphasized transfer learning as a key factor in boosting accuracy, reducing training time, and improving generalization, thereby making next-generation architectures like EfficientNet-B5 highly suitable for developing reliable diagnostic models in medical imaging applications.

Rasheed et al. [26] proposed a CNN-based framework for brain tumor classification that uses specialized image improvement techniques, including Gaussian blur sharpening and Contrast-Limited Adaptive Histogram Equalization (CLAHE). The authors designed a lightweight CNN architecture featuring skip connections, batch normalization, and dropout regularization. The model was trained using a merged dataset of 7023 MRI images from BR35H, Figshare, and SARTAJ, divided into four sections: Pituitary, Glioma, Meningioma, and Non-Tumor [16,19,25]. The proposed method achieved an accuracy of 97.84%, surpassing pre-trained architectures such as VGG16, ResNet50, and InceptionV3 in both classification accuracy and computational efficiency. This study shows that using customized image preprocessing along with lightweight CNN architectures can be effective and achieve high diagnostic accuracy with fewer parameters, making it more practical for real-world clinical use.

Miah et al. [27] proposed a hybrid brain tumor detection framework that combines unsupervised clustering-based feature extraction with CNN classifiers. The study used a real-world MRI dataset consisting of 1892 images from 153 patients, including both healthy individuals and those with diagnosed brain tumors. Before classification, unsupervised clustering was applied to improve the quality of extracted features. All images were resized from their original (512 × 512) dimensions to (227 × 227) pixels during preprocessing to match CNN input requirements. Three classification strategies were evaluated: CNN with SoftMax, CNN with Radial Basis Function (RBF), and CNN with Decision Tree (DT). Among these, CNN with SoftMax delivered the best results, achieving an accuracy of 99.32%, precision of 98.83%, sensitivity of 100%, and specificity of 96.44%. This study demonstrates that integrating advanced clustering techniques with DL can significantly enhance diagnostic accuracy and reliability in medical image classification.

Nassar et al. [28] proposed a hybrid DL model for brain tumor classification that employs majority voting across five fine-tuned CNN architectures: AlexNet, GoogLeNet, NASNet-Mobile, ShuffleNet, and SqueezeNet (all in their standard pre-trained ImageNet versions). The system was trained and validated using a publicly available T1-Weighted Contrast-Enhanced (T1W-CE) MRI dataset, which includes 3064 images from 233 patients. This ensemble model achieved an accuracy of 99.31% with minimal preprocessing, demonstrating the robustness of multi-model hybridization and majority voting in handling tumor heterogeneity. Additionally, it reached a sensitivity of 98.30%, indicating its potential as a dependable assistive tool for radiologists in clinical decision-making.

Disci et al. [29] proposed a transfer learning-based framework utilizing six pre-trained CNN architectures: Xception, MobileNetV2, InceptionV3, ResNet50, VGG16, and DenseNet121. In this study for multiclass classification, they used a dataset of brain MRI scans divided into four categories: Glioma, Meningioma, Pituitary Tumor, and Non-Tumor. The models were trained on a publicly available, balanced dataset consisting of 7023 images [21] and evaluated using accuracy, precision, recall, and F1-score. Xception achieved the highest weighted accuracy of 98.73% among the tested models. To improve model robustness, they employed data augmentation and Region of Interest (RoI) cropping techniques. Although the model classified Non-Tumor and Pituitary Tumor cases with high recall, it showed relatively lower recall for Glioma and Meningioma, reflecting the difficulty in differentiating tumors with overlapping visual characteristics. The study further emphasized the trade-off between interpretability and predictive performance in DL-based medical image classification. Overall, the work illustrates the potential of adapting pre-trained CNNs for clinical diagnostic tasks through appropriate fine-tuning and preprocessing strategies.

Badza and Barjaktrovic [30] used a lightweight CNN for brain tumor classification using T1W-CE MRI scans. This architecture comprises 22 layers, which include convolutional, dropout, and pooling blocks. This model was trained on BR35H with 3064 images, resulting in an augmented dataset of 9192 images. Validation was performed using 10-fold cross-validation schemes. The model achieved a maximum accuracy of 97.39%, while the more clinically relevant subject-wise validation achieved an accuracy of 88.48% on the augmented dataset. These findings demonstrate the model’s robustness in handling previously unseen patient data, indicating its suitability for clinical environments with constrained computational capacity.

Athisayamani et al. [31] proposed a complete study on a DL pipeline for multiclass brain tumor classification using MRI images. Their approach integrated adaptive preprocessing, segmentation, and optimization-enhanced feature extraction. Specifically, they used an Adaptive Canny Mayfly Algorithm (ACMA) for edge detection, followed by feature extraction using the Spatial Gray Level Dependence Matrix (SGLDM) and dimensionality reduction through a modified Chimp Optimization Algorithm (EChOA). This biologically inspired pipeline was designed to efficiently reduce redundancy while preserving important spatial features. ResNet152 served as the backbone of their DCNN, with a Softmax layer for classification. The framework was trained and validated on datasets [24,32,33], including Figshare, BraTS 2019, and MICCAI, achieving an overall accuracy of 98.85%, with precision and recall scores of 96.81% and 97.64%, respectively. The proposed method outperformed baseline models such as standard CNNs, linear Support Vector Machines (SVMs), and ensemble classifiers. These findings emphasize the importance of integrated optimization and advanced feature engineering in building robust diagnostic models. This study demonstrates how biologically inspired optimization techniques combined with deep feature extraction can enhance diagnostic accuracy and reliability across diverse brain tumor types.

Zahoor et al. [34] developed Res-BRNet, a specialized CNN architecture for brain tumor classification using MRI images. This model integrates deep residual learning with boundary-aware feature extraction, incorporating custom spatial blocks to highlight tumor edges and residual blocks to capture both local and global texture features. The architectural design was particularly suited to handling subtle structural variations often observed in complex tumor morphologies. The authors trained and validated their model on a dataset of 7872 MRI scans, compiled from the Kaggle, BR35H, and Figshare repositories. All images were resized to match the network’s input requirements. Res-BRNet achieved outstanding performance, with an accuracy of 98.22%, precision of 98.22%, sensitivity of 98.11%, and an F1-score of 98.41%, surpassing several state-of-the-art CNN baselines. The model’s robustness across heterogeneous datasets underscores its potential for real-world deployment. The study demonstrates that combining residual learning with spatially adaptive, boundary-focused feature extraction significantly enhances the classification accuracy of brain tumor diagnostics.

Abdusalomov et al. [35] proposed a framework leveraging YOLOv7 for multiclass brain tumor detection using enhanced MRI data. The model incorporates advanced components such as the Convolutional Block Attention Module (CBAM), Bi-directional Feature Pyramid Network (BiFPN), and Spatial Pyramid Pooling–Fast (SPPF+) to enhance feature representation and detection accuracy. These modules collectively improve spatial localization and enable multi-scale feature refinement, which is crucial for small or ambiguous tumor boundaries. The study utilized curated datasets Figshare and SARTAJ [19,25] with 10,288 MRI images, which was further expanded to 51,448 images through Gaussian filtering, normalization, and extensive data augmentation. The proposed framework achieved an accuracy of 99.55%, demonstrating its effectiveness in precise tumor classification. The adoption of YOLOv7, typically used in general object detection, shows promise when adapted for medical imaging tasks. This work highlights the potential of integrating state-of-the-art object detection architectures with domain-specific optimizations to deliver reliable and robust brain tumor diagnostics.

Compared to the related studies summarized in Table 1, our ensemble model achieves better performance in both architectural design and functional performance over prior models. While some approaches [15,17] primarily aim to optimize single-CNN architectures through hyperparameter tuning, others [20,26] employ dual CNNs or ensemble voting mechanisms but lack integration of gradient boosting or meta-learning techniques, constraining their learning capability for diverse and complex tumor features. Some methods [30,35] incorporate clustering-based preprocessing, while others [20] apply segmentation techniques to improve tumor localization. In contrast, our model used three advanced CNN architectures, EfficientNetB7, InceptionV3, and Xception, and enhanced prediction performance by stacking their output using LightGBM. This ensemble technique enables the model to capture both spatial and semantic variations while accurately capturing non-linear dependencies between ensemble outputs. By incorporating LightGBM, the model achieves higher classification efficiency and interpretability, supporting its applicability as a robust and scalable solution for clinical brain tumor detection. Furthermore, while earlier studies [22] relied on a single interpretability method like Grad-CAM, our framework implements a more comprehensive suite of explainability methods, including Grad-CAM++, SHAP, and LIME, thereby improving interpretability without compromising accuracy.

## 3. Proposed Explainable Deep Ensemble Meta-Learning Model

**Figure 1:**
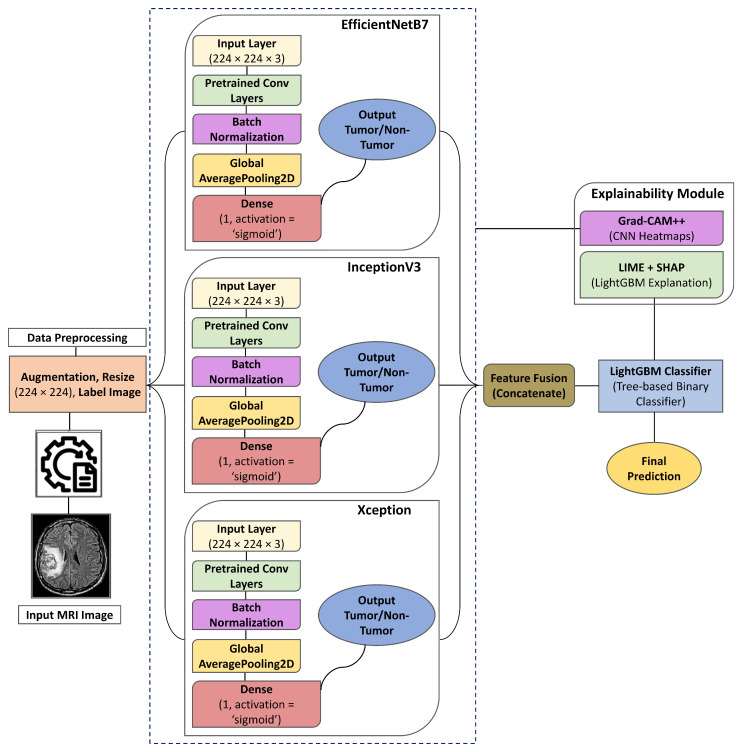
Overview of the proposed Explainable Deep Ensemble Meta-Learning model architecture. MRI images undergo preprocessing and are independently processed through EfficientNetB7, Xception, and InceptionV3. The extracted features from each model are concatenated and passed to a Light Gradient Boosting Machine (LightGBM) classifier to generate the final prediction.

The proposed model adopts a comprehensive and systematic approach to develop a robust and interpretable framework for brain tumor detection in MRI image as illustrated in Figure 1. It integrates pre-trained DL architectures, ensemble learning strategies, and XAI techniques to enhance both performance and transparency. By leveraging EfficientNetB7, InceptionV3, and Xception models for feature extraction, the framework benefits from reduced training time and improved generalization, eliminating the need for training from scratch. The combination of advanced architectures with interpretability methods ensures that the system not only delivers accurate predictions but also provides meaningful insights into the model’s decision-making process.

In the proposed framework, feature fusion is performed at the output probability level rather than the raw feature space. Specifically, each backbone network, EfficientNetB7, InceptionV3, and Xception produces a single probability score for the “tumor” class after its respective classification layer. These three scalar probability values are then concatenated directly to form a three-dimensional meta-feature vector $\left[p_{\mathrm{Eff}},p_{\mathrm{Inc}},p_{\mathrm{Xcp}}\right]$ and $\left[p_{Eff},p_{Inc},p_{Xcp}\right]$. Since all values are probabilities in the range [0, 1], no additional normalization, alignment, or dimensionality reduction is required prior to fusion. This fusion occurs after each backbone’s global average pooling and dense classification stage (i.e., in the output space), ensuring that the meta-learner receives uniformly scaled, semantically aligned inputs. The resulting meta-feature vector is then passed to the LightGBM classifier, which models non-linear relationships between backbone outputs to produce the final prediction.

### 3.1. Dataset Collection

**Figure 2:**
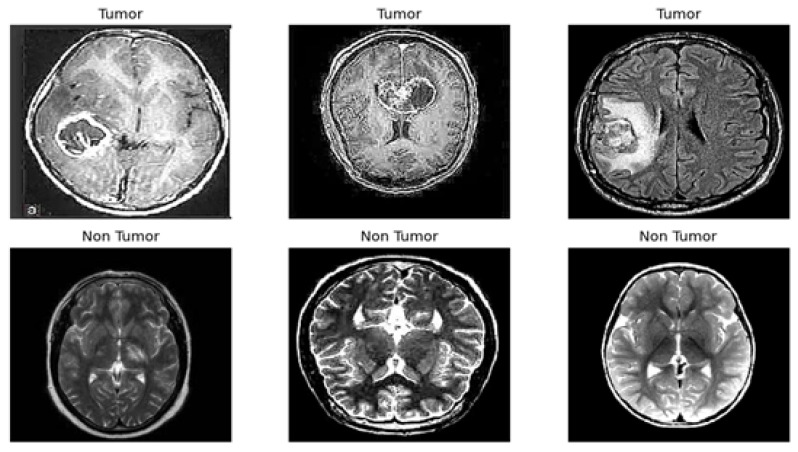
A visual representation of tumor and non-tumor MRI scans.

We utilized the publicly available BR35H Brain Tumor Detection 2020 dataset [16], selecting 3000 MRI images for training and testing purposes. An additional 60 images, which were not used during training, were reserved for independent model evaluation. Notably, we excluded the BR35H-Mask-RCNN segmentation subset and focused exclusively on the classification task. The labeled portion is evenly divided between the tumor and non-tumor classes, resulting in a balanced dataset that is well-suited for both training and evaluation. Representative images are presented in Figure 2, and a detailed summary of the dataset is provided in Table 2. The dataset was selected due to its clarity, consistent annotations, and suitability for training both individual base models and ensemble frameworks in medical image classification tasks.

### 3.2. Data Preprocessing

Data preprocessing is a critical initial phase in developing DL models, particularly in medical imaging, where the complexity and variability of input data can significantly influence model performance. Unlike general DL tasks involving simple format conversions, preprocessing in medical imaging requires deeper standardization to ensure data consistency and enable NNs to effectively learn complex and subtle features.

The first preprocessing step is normalization, which standardizes pixel intensity values across the dataset. In this study, normalization is performed by dividing each pixel value by 255, thereby scaling all values to the range [0, 1]. This bounded range reduces numerical instability and enhances both the efficiency and stability of gradient-based optimization during training, making it easier for DL models to learn effective mappings between inputs and outputs.

Next, all images are resized to a fixed dimension of (224×224×3) to ensure compatibility with widely used pre-trained CNN architectures such as EfficientNetB7, InceptionV3, and Xception. This standardized input size maintains structural consistency and enables seamless integration of pre-trained feature extractors, ensuring architecture.

To ensure data integrity and eliminate duplicates prior to dataset splitting, each imange $x\;\in R^{H\;\times \;W\;\times \;C}$ was resized to (224×224×3) and passed through a SHA-1 hash function. This cryptographic function, defined as $x\in R^{H\times W\times C},\;h=\mathrm{SHA}1\left(\mathrm{bytes}(x)\right)$, produces a unique 160-bit hash $h\in {\left\{0,1\right\}}^{160}$ for each image. Identical hashes indicate duplicate image content, which was removed to prevent data leakage across the training, validation, and test sets, thereby improving the fairness and reliability of performance evaluation.

**Figure 3:**
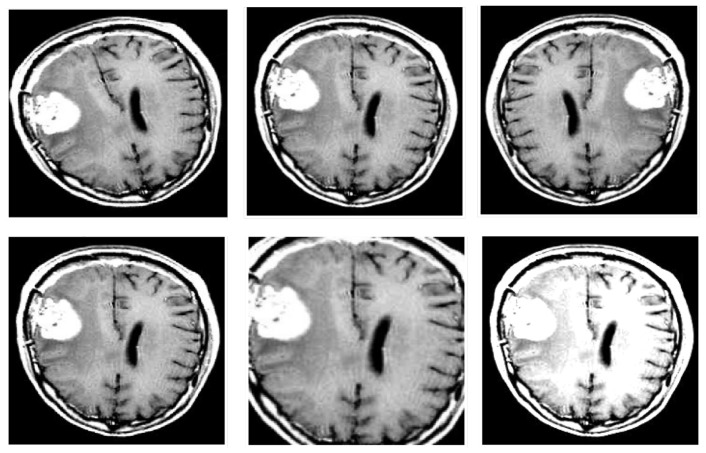
Data augmentation examples on tumor MRI scans, including rotation, flip, brightness shifts, and zoom.

A comprehensive data augmentation pipeline was employed to improve model robustness and address the limited sample diversity inherent in medical imaging datasets. The original training set of 1799 images was expanded to 12,593 samples through the application of various augmentation techniques. These included random rotations within ±20 degrees, horizontal and vertical flipping, controlled brightness adjustments, and zoom transformations with scaling factors ranging from 0.8 to 1.2. Mathematically, this process can be expressed as $x_{avg}=A\left(x,\sigma \right)$, where *A* represents the augmentation function, *x* is the original input image, and σ denotes the set of augmentation parameters. These augmentation strategies (Figure 3) simulate a variety of real-world conditions and anatomical variations, which in turn improve the model’s ability to generalize to new, unseen data. Overall, the combination of normalization, resizing, and augmentation establishes a robust preprocessing pipeline that enhances model training and contributes to reliable and accurate brain tumor classification.

An essential component of the preprocessing phase involves addressing class balance. Unlike many real-world medical datasets [32,33] that suffer from class imbalance, the dataset used in this work is inherently balanced, comprising 1500 MRI images labeled as tumors and 1500 labeled as non-tumors. As a result, no additional balancing techniques such as oversampling, undersampling, or class reweighting were required. To prepare the data for model development, the dataset was partitioned into three subsets: train, test, and validation. Stratified sampling (60/20/20) was applied during this split to ensure that the class distribution remained consistent across all subsets. This strategy helps reduce sampling bias and supports more reliable evaluation of model performance.

**Figure 4:**
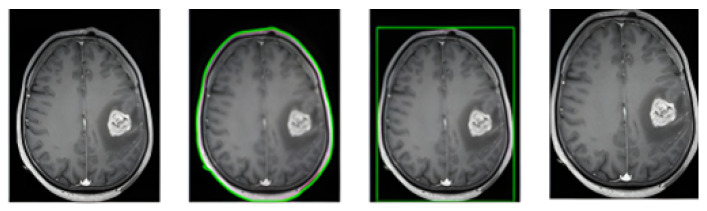
Automated brain region extraction steps using cropping method: original image, contour detection, Region of Interest (RoI) bounding, and final cropped output. The green contour marks the detected boundary, with RoI used for downstream classification.

A crucial preprocessing step specific to this study is the isolation of the brain region from each raw MRI image before it is passed into the classification framework. This step ensures that the model concentrates solely on the relevant anatomical structures, excluding irrelevant background pixels that may introduce noise and hinder learning. Such focused preprocessing enhances the model’s ability to detect meaningful patterns in the data, contributing to improved prediction accuracy. To implement this, a contour-based cropping method was used. The method works by automatically identifying and extracting the RoI which corresponds to the brain tissue in each MRI slice presented in Figure 4. It detects the largest external contour in the image, assuming this represents the primary anatomical structure of interest. As a fully automated process, it eliminates the need for manual intervention in defining ground truth regions, thereby streamlining the data preparation pipeline. This automation not only improves efficiency but also ensures consistency and scalability when training the classification model across large datasets.

### 3.3. Feature Extraction

In this study, three high-performing pre-trained CNN models, EfficientNetB7, InceptionV3, and Xception, are employed for feature extraction. Each model brings distinct strengths in capturing complex visual patterns and offers robust representations well-suited for medical imaging tasks. Transfer learning is applied by initializing these CNNs with ImageNet-pretrained weights and then fine-tuning them on the target MRI dataset, allowing the models to adapt to domain-specific features while benefiting from previously learned general visual knowledge.

The extracted features from each of these CNNs are then used as input to a LightGBM [36]. This two-stage approach decouples feature extraction from the classification process, enabling efficient computation and streamlined model training. The linear classifier is trained to make the final classification decisions based on the rich and diverse feature embeddings produced by the CNNs. This method capitalizes on the representational power of the DCNN architecture while maintaining computational simplicity during classification. The overall process is further formalized through the mathematical representation provided in Equation (1).

(1) $${⌀}_{i}=f_{{\mathrm{CNN}}_{i}}\left(x_{\mathrm{avg}}\right)$$

where (normal/italic/bold/subscript/superscript). ${⌀}_{i}$ is the extracted feature vector and $f_{{CNN}_{i}}$ represents the CNN model.

The extracted feature vectors encapsulate critical attributes such as tumor boundaries, texture patterns, and other structural details essential for accurate classification. To enhance the stability and efficiency of the feature extraction process, batch normalization was incorporated into the intermediate layers of the network during training. This technique helps maintain consistent distribution of activations, accelerating convergence and reducing internal covariate shift. In addition, L2 weight regularization was employed to discourage the network from assigning excessively large weights to specific parameters, thereby mitigating the risk of overfitting and promoting better generalization. Following the feature extraction phase. The model outputs from each CNN are combined into a meta-feature vector, which is then passed to the LightGBM classifier for the final prediction. This layer integrates two stacked Multiple-Instance Learning (MIL) models—an advancement over the earlier prototype, which utilized only a single MIL model. The stacking strategy allows for deeper and more nuanced interpretation of localized features across instances within each image. The applied regularization during this phase is quantified through the regularization loss, $L_{reg}$ defined in Equation (2).

(2) $$L_{\mathrm{reg}}=\lambda \sum_{j}w_{j}^{2}$$

where λ is the regularization parameter and $w_{j}^{2}$ is the model weights.

We utilized Optuna for automated hyperparameter optimization to further enhance the performance of the meta-learner. Specifically, the LightGBM classifier was tuned over 25 trials using Bayesian optimization with five-fold stratified cross-validation applied to the meta-feature validation set. The best configuration identified was as follows: num_leaves = 50, learning_rate = 0.0052, n_estimators = 187, min_child_samples = 14, reg_alpha = 0.66, reg_lambda = 0.95, subsample = 0.83, and colsample_bytree = 0.77. These parameters were used to retrain the final LightGBM model on the full meta-validation set, which was subsequently evaluated on a held-out test set. Reporting these details improves reproducibility and supports transparency in the ensemble construction process.

### 3.4. Ensemble Learning and Meta-Learner Integration

To improve prediction accuracy, an ensemble learning strategy was employed by integrating the outputs of three CNN models. These model outputs were combined using a soft voting mechanism, which averages the class probability predictions of each model to generate a final classification decision. This approach leverages the complementary strengths of the individual models, allowing them to compensate for each other’s limitations and produce a more robust and reliable ensemble prediction.

LightGBM was introduced as a meta-learner to further strengthen the framework. In this setup, the probability outputs from the individual CNN models serve as input features for LightGBM [36]. Known for its effectiveness in handling structured/tabular data and optimizing classification tasks, LightGBM operates as an advanced gradient boosting framework capable of efficiently training over large numbers of boosting iterations. Its ability to manage complex decision boundaries and scale to large feature spaces makes it particularly suitable as a refinement stage for ensemble outputs. The overall ensemble prediction is formulated in Equation (3), while the enhanced, final predictions refined through LightGBM are defined in Equation (4).

(3) $$P_{\mathrm{ensemble}}=\frac{1}{N}\sum_{i=1}^{N}P_{{\mathrm{CNN}}_{i}}$$

where $P_{ensemble}$ represents the aggregated probability and $P_{{CNN}_{i}}$ is the probability output of the *i*-th CNN model. Soft voting enhances the robustness and reliability of predictions by utilizing diverse model perspectives.

(4) $$P_{\mathrm{final}}=f_{\mathrm{LightGBM}}\left(P_{\mathrm{ensemble}}\right)$$

The integration of CNNs with LightGBM delivers both high accuracy and robust performance. Beyond improving predictive capability, LightGBM contributes to model transparency through its feature importance analysis, offering valuable insights into which features most strongly influence classification outcomes. This enhances the overall interpretability and trustworthiness of the framework.

### 3.5. Explainable Artificial Intelligence Integration

The model includes three complementary XAI techniques, Grad-CAM++, LIME, and SHAP, which together offer different strengths of explanation. Their selection was guided by their specific interpretability contributions. Grad-CAM++ allows us to perform spatial localization MRI scans and mark the relevant regions that influence the model’s decision. LIME provides explanations for the particular and instance level for the input provided, which helps focus on specific details. SHAP explains global interpretability by taking all the outputs of the base CNN models with ensemble output and calculating each base model’s contribution, thus interpreting globally. This combined approach not only improves reasoning but also brings the model’s decision-making processes more in line with human clinical judgment and trust in AI-assisted diagnostics while strengthening explainability and transparency.

Grad-CAM++ was incorporated to ensure both transparency and clinical relevance. It generates class activation maps by assigning importance weights to the gradients of the predicted class score with respect to the outputs of convolutional layers [7]. This process highlights the regions in the input image that most strongly influence the model’s decision, as defined in Equation (5).

(5) $$L_{\mathrm{GradCAM}++}=\sum_{k}\alpha_{k}\cdot R\left(\frac{\partial y^{c}}{\partial A_{ij}^{k}}\right)$$

where $\alpha_{k}$ are weights, $y^{c}$ is the class score, and $A_{ij}^{k}$ represents activation maps. These maps are overlaid on the original images, creating heatmaps that highlight the regions most influential in the model’s decision-making process. This interpretability empowers clinicians to validate model predictions and build trust in the AI system.

LIME was incorporated to ensure interpretability and clinical relevance. It works by generating perturbed samples around a prediction instance and fitting an interpretable surrogate model that approximates the behavior of the complex classifier within a localized region. This approach enables clinicians to understand which input features were most influential for a specific prediction [10]. Formally, LIME constructs the surrogate model g∈G by minimizing a loss function L that measures the fidelity of *g* in approximating the original model *f* in the locality $\pi_{x}$ while simultaneously penalizing the complexity of *g*. This optimization objective is defined in Equation (6).

(6) $$\xi \left(x\right)=\underset{g\in G}{\arg\min}\;L\left(f,g,\pi_{x}\right)+\Omega \left(g\right)$$

In the context of this study, LIME is applied to the output probabilities of the base CNN models passed to the LightGBM meta-learner. The surrogate model g(z) is expressed as a sparse linear model fitted to the locally weighted data. This interpretable approximation of the model’s behavior is defined in Equation (7).

(7) $$f\left(x\right)\approx g\left(z\right)=\sum_{i=1}^{M}\beta_{i}\cdot z_{i}$$

SHAP was integrated into the proposed model to enhance the global interpretability of the classification decisions produced by the LightGBM meta-learner. SHAP operates based on cooperative game theory and assigns each feature a Shapley value, which quantifies its individual contribution to the final prediction. In this study, the input to LightGBM comprises class probability scores generated by the CNN models. By calculating the marginal contribution of each CNN output, SHAP identifies which model contributes most significantly to the ensemble’s final classification. The Shapley value $\phi_{i}$ for a feature is defined in Equation (8).

(8) $$\phi_{i}=\sum_{S\subseteq F\setminus \{i\}}\frac{|S|!\left(|F|-|S|-1\right)!}{|F|!}\left[f(S\cup \{i\})-f(S)\right]$$

### 3.6. Training and Hyperparameter Optimization

The training process was guided by the Binary Cross-Entropy (BCE) loss function, which is ideal for binary classification tasks. BCE calculates the difference between the true labels and predicted probabilities, as defined in Equation (9). Optuna was employed to optimize key hyperparameters, including learning rate (10^−5^, 10^−2^), batch size (16, 32, 64), and dropout rate (0.1, 0.5). In each trial, Optuna evaluated a specific combination of these parameters by training the model and computing the validation loss, as formulated in Equation (9), ultimately selecting the configuration that achieved the lowest loss.

(9) $$L_{\mathrm{BCE}}=-\frac{1}{N}\sum_{i=1}^{N}\left[y_{i}\log\left({\hat{y}}_{i}\right)+\left(1-y_{i}\right)\log\left(1-{\hat{y}}_{i}\right)\right]$$

where $y_{i}$ is the true label, ${\hat{y}}_{i}$ is the predicted probability, and *N* is the total number of samples.

(10) $$\mathrm{Objective}=\min_{\theta }L_{\mathrm{BCE}}$$

where θ represents the set of hyperparameters being tuned. This automated process ensured optimal parameter selection, balancing accuracy and computational efficiency.

## 4. Performance Evaluation

The proposed model is evaluated using a comprehensive set of metrics, such as accuracy, precision, recall, and Area Under the Receiver Operating Characteristic (ROC) curve (AUC), to ensure its reliability and decision-making quality. XAI methods were integrated to gain deeper insight into the model’s behavior during inference.

### 4.1. Experiment Setup

The model is trained and evaluated using a balance dataset BR35H of 3000 MRI images, which is equally divided into tumor and non-tumor classes. A stratified split was applied to maintain class distribution, with 60% of the data used for training, 20% for validation, and 20% for testing. All experiments were conducted on a system equipped with an NVIDIA RTX 3070 GPU (8 GB VRAM), 32 GB of RAM, and Python-based libraries (Python 3.12), including TensorFlow, LightGBM, and OpenCV. The performance metrics used for evaluation are as follows:

**Accuracy:** Accuracy is the percentage of correct predictions among all predictions. It provides a general indication of the model’s overall performance defined in Equation (11), where TP is the true-positive and TN is the true-negative predictions.

(11) $$\mathrm{Accuracy}=\frac{TP+TN}{\mathrm{Total}\;\mathrm{No}.\;\mathrm{of}\;\mathrm{Samples}}$$

**Precision:** It is the model’s ability to correctly identify positive cases derived in Equation (12), where FP is the false-positive predictions. High precision means a low rate of false prediction.

(12) $$\mathrm{Precision}=\frac{TP}{TP+FP}$$

**Recall:** It gauges the model’s capability to recognize every positive case within the data. It describes the ability of the model to retrieve relevant cases defined in Equation (13), where FN is the false-negative predictions. High recall indicates that the model has successfully identified most of the true-positive cases with minimal FNs.

(13) $$\mathrm{Recall}=\frac{TP}{TP+FN}$$

**F1-Score:** It provides a balanced measure that considers both FPs and FNs defined in Equation (14). The F1-score is particularly helpful when there is an imbalance within the dataset since it prevents the scenario where either precision or recall overshadows the assessment.

(14) $$F1\text{-}\mathrm{Score}=\frac{2\;(\mathrm{Precision}\times \mathrm{Recall})}{\mathrm{Precision}+\mathrm{Recall}}$$

**AUC-ROC:** It assesses the performance of a model on a use case for any classification problem by assuming various thresholds for classification and calculating the ability to separate the classes derived in Equations (15) and (16) to measure the True-Positive Rate (TPR) and False-Positive Rate (FPR), respectively. A model with higher scores in AUC is assumed to perform better in the ranking of positive instances in comparison to negative instances. It is especially useful for imbalanced datasets.

(15) $$\mathrm{TPR}=\frac{TP}{TP+FN}$$

(16) $$\mathrm{FPR}=\frac{FP}{FP+TN}$$

### 4.2. Results Analysis

**Figure 5:**
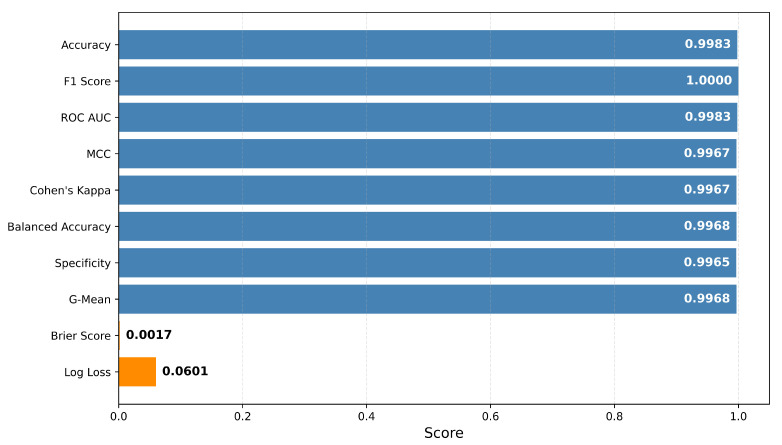
Comprehensive evaluation metrics of the proposed model, including accuracy, F1-score, AUC-ROC, MCC, Cohen’s Kappa, balanced accuracy, specificity, G-mean, Brier score, and log loss.

The performance of the proposed model was evaluated against rigorous classification benchmarks, as shown in Figure 5. The model performed well on all primary and secondary metrics, suggesting its usefulness and dependability for clinical diagnosis applications. In particular, the model reported a 99.83% accuracy, an F1-score of 1.000, and an AUC-ROC of 0.9948 which states to its remarkable performance in classification tasks. Further evaluation metrics, such as the Matthews Correlation Coefficient (MCC) and Cohen’s Kappa, which both equal κ=0.9967, demonstrate strong concordance between predictions and actual labels, even in the presence of imbalance and noise. Balanced accuracy (0.9968), specificity (0.9965), and G-mean (0.9968) also underscore the model’s unbiased performance in detecting both classes. Moreover, log loss (0.0601), together with a Brier score of 0.0017, illustrates that the model’s probabilistic outputs are well calibrated and that the model is confident; 95% Confidence Limits (CLs) are reported throughout: test accuracy 99.83% (599/600; 95% Wilson CI: 99.06–99.97%). The model achieved 99.83% accuracy on a separate 20% test set (n=600), with near-perfect precision, recall, and F1-score. The LightGBM meta-learner was tuned and evaluated via five-fold stratified cross-validation on the training/validation portion of the data (80% of the total dataset) using an out-of-fold stacking strategy. In each fold, probability outputs from the CNN backbones were generated for the validation split and subsequently used to train the meta-learner, ensuring no leakage between folds. The five-fold cross-validation accuracies were 99.50%, 99.67%, 99.67%, 99.83%, and 99.83%, yielding a mean accuracy of 99.70% ±0.13%. Mean precision, recall, and F1-score across folds were all ≥0.996, confirming that the high test-set performance is consistent and not dependent on a particular split. Cross-validation results are reported separately from independent test performance.

**Figure 6:**
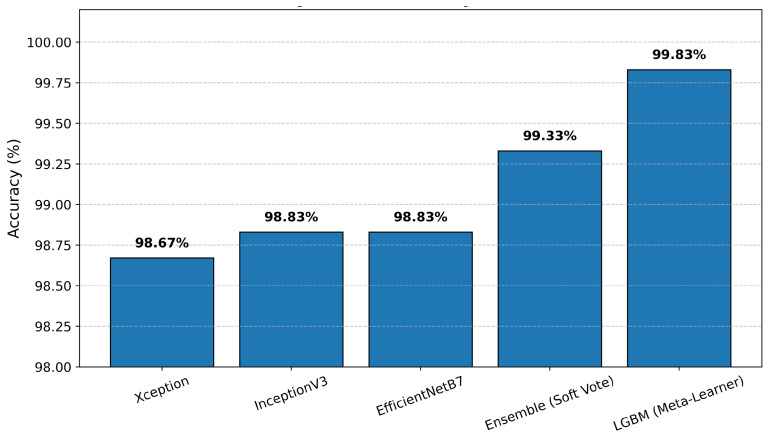
Ablation study showing the individual and combined performance of different CNN backbones (Xception, InceptionV3, EfficientNetB7), ensemble (soft voting), and the final LightGBM meta-learner. The LGBM approach achieves the highest test accuracy (99.83%), outperforming all individual models and ensemble methods.

**Ablation Study and Component Contributions:** Individually, the backbones achieve 98.67% (Xception), 99.00% (InceptionV3), and 98.83% (EfficientNetB7) on the held-out test set (n=600), as illustrated in Figure 6. Simple probability averaging improves accuracy to 99.33%. Replacing the average with a LightGBM meta-learner further increases accuracy to 99.83%.

Building upon the ablation study results, the final evaluation compared the fully configured LGBM meta-learner with the best-performing baseline CNN on the held-out test set (n=600). The LGBM achieved an accuracy of 99.83% (599/600 correct; 95% Wilson CL: 99.06–99.97%), while the baseline CNN achieved 99.00% (594/600 correct; 95% Wilson CL: 98.06–99.64%), representing a numerical improvement of +0.83%. McNemar’s test on paired predictions indicated that this difference was not statistically significant at the 0.05 level (*p* > 0.05). Nonetheless, the LGBM outperformed the baseline across all primary metrics accuracy, precision, recall, and AUC—indicating a more stable and reliable predictive capability. These gains were achieved without compromising generalization, as evidenced by well-aligned training and validation curves and the use of early stopping to mitigate overfitting. We therefore interpret the LGBM’s higher accuracy as a robust and reproducible trend within the 95% CL, rather than definitive statistical superiority.

**Figure 7:**
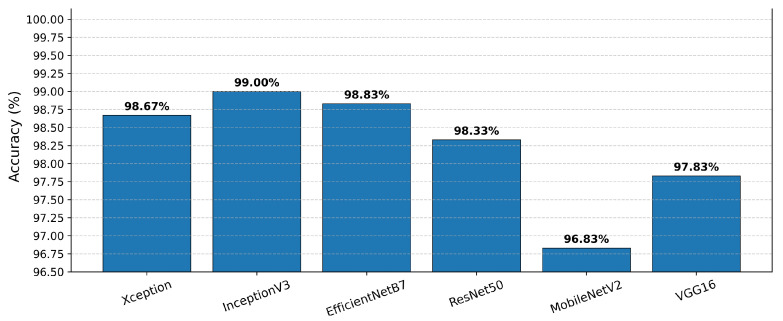
Comparative accuracies of baseline CNN models (Xception, InceptionV3, EfficientNetB7, ResNet50, MobileNetV2, and VGG16).

We re-implemented three widely used state-of-the-art CNN architectures, ResNet50, MobileNetV2, and VGG16, on the same dataset using identical preprocessing, aug-mentation, and evaluation protocols as applied to our proposed method to further ensure a fair comparison. As shown in Figure 7, ResNet50 achieved 98.33% accuracy, MobileNetV2 achieved 96.83%, and VGG16 achieved 97.83%. All three baselines performed lower than the LGBM meta-learner’s 99.83% accuracy, underscoring the superior predictive performance and robustness of our approach. This consistent improvement over both lightweight architectures (e.g., MobileNetV2) and deeper architectures (e.g., VGG16, ResNet50) demonstrates the adaptability and effectiveness of the proposed framework across a range of model complexities.

**Figure 8:**
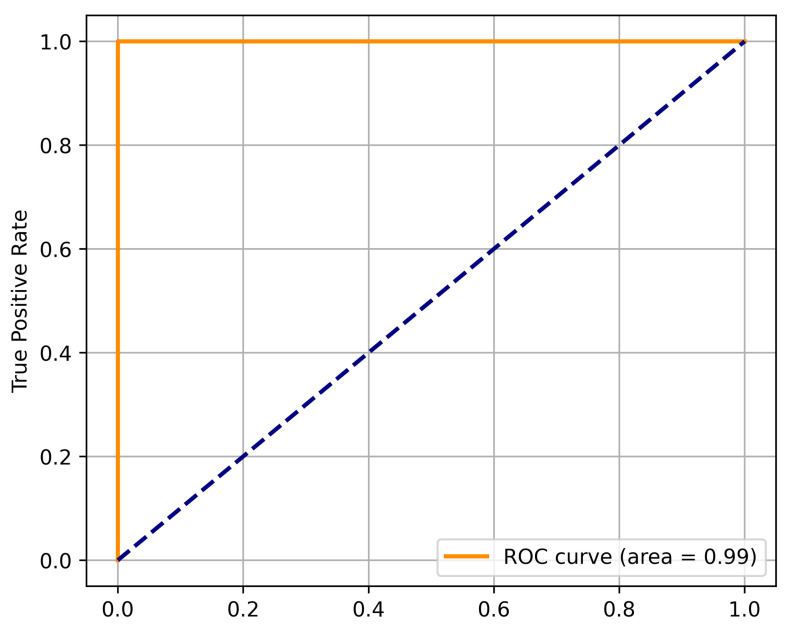
Receiver Operating Characteristic (ROC) curve of the proposed model.

The accuracy of the proposed model in performing the discrimination tasks was evaluated using ROC curves, as shown in Figure 8. The ROC is one of the most common techniques to evaluate the performance of binary classifiers and offers insight into the relationship between sensitivity (or TPR) and 1-specificity (FPR) for some threshold level. The ensemble model achieved an AUC of 0.9948, which is an indication of improved performance in distinguishing the tumor from non-tumor MRI scans. This near-perfect AUC value suggests that the model is able to discriminate between the two classes with a higher sensitivity and incredibly lower FPs across a wide range of threshold settings. The accelerated increase and the curve’s proximity to the upper left corner demonstrate the exceptionally low FPR, which is critical in clinical settings where unnecessarily diagnosing conditions can lead to distress and unnecessary procedures for patients. The model exhibited superior performance in terms of accuracy, which indicates strong generalization and robustness in the presence of uncertain boundaries in the problem, which reinforces the readiness of the model for application in real-life medical scenarios. Thus, ROC analysis shows that the ensemble framework provides trusted predictions and therefore can be further developed for use in clinical decision support systems.

**Figure 9:**
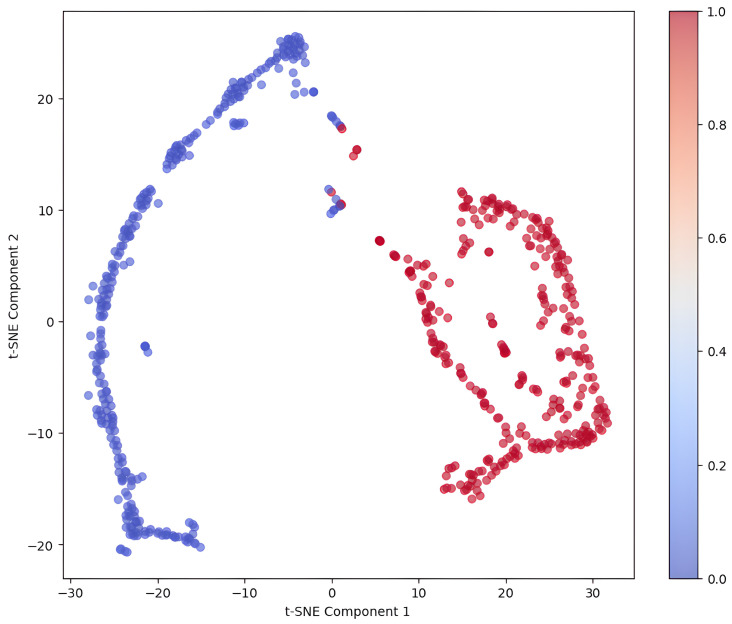
t-SNE of the CNN–LightGBM ensemble’s learned meta-features. Tumor and non-tumor classes form distinct clusters in the 2D projection, confirming the model’s capability to generate separable and clinically meaningful internal representation.

To examine the internal structure of the learned feature representations, a t-distributed Stochastic Neighbor Embedding (t-SNE) technique was applied to the ensemble of CNN models and was then refined by the LightGBM meta-learner to reduce the dimensionality of the meta-feature space. As illustrated in Figure 9, the two-dimensional projection identifies two distinct, compact clusters that are well separated spatially and correspond to the tumor and non-tumor MRI images. This remarkable separability in the latent space demonstrates that the model captures class-specific patterns of features with intra-class uniformity. The absence of overlap between the clusters suggests strong inter-class variance has been achieved, coupled with effective representational learning, which is important in medical classification problems since clarity of latent embedding in the model has a direct relationship with confidence in the decisions made. The visualization proves the model successfully encodes the relevant pathological features and aligns its latent reasoning with the clinically meaningful differences. Thus, the t-SNE results validate the effectiveness of the proposed pipeline for feature extraction while simultaneously providing justification for model interpretability and generalizability.

**Figure 10:**
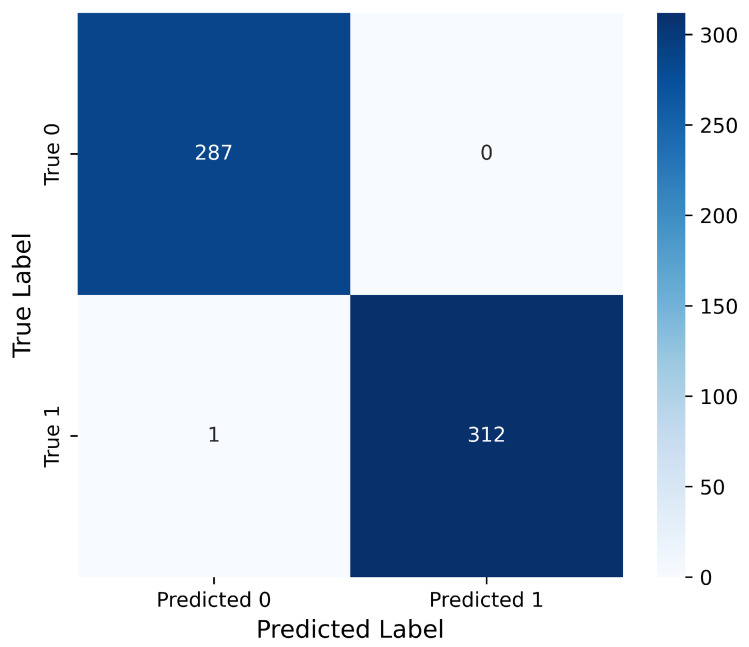
Confusion matrix of the proposed model on the test dataset. The model correctly classified 287 non-tumor and 312 tumor cases, with only one false negative (FN) prediction observed. This indicates a remarkably high sensitivity (recall) and specificity, essential for clinical applicability.

A confusion matrix approach to assessing model performance provides more insight into a model’s predictive power than accuracy alone. This insight becomes even more valuable in the context of medical image classification, where TPR, FNR, and specificity relative to model performance across both classes are of critical importance. While it is essential that the model achieves high accuracy, the actual classification results must be evaluated to determine the model’s clinical acceptability and safety. In this regard, the confusion matrix emerges as an important means to assess not just validation but validation with respect to errors that directly impact the diagnostic process. As shown in Figure 10, the confusion matrix illustrates the specifics of the model’s classification accuracy as well as errors with deep insights. Out of 600 test samples, there was only one FN error where a tumor case was predicted as non-tumor; all other predictions were made correctly. This gives a sensitivity (recall) of 99.68%, indicating that the model efficiently detects actual tumor cases with a high probability, and a specificity of 100%, meaning all non-tumor cases were correctly predicted without errors. Such a sensitive and specific diagnosis in clinical practice is important because it decreases the chances of FN results that could delay necessary treatment, while also avoiding FPs that may cause unnecessary stress or medical procedures. These findings demonstrate the accuracy and dependability of the ensemble system framework in reliably distinguishing between tumor and non-tumor MRI scans.

**Figure 11:**
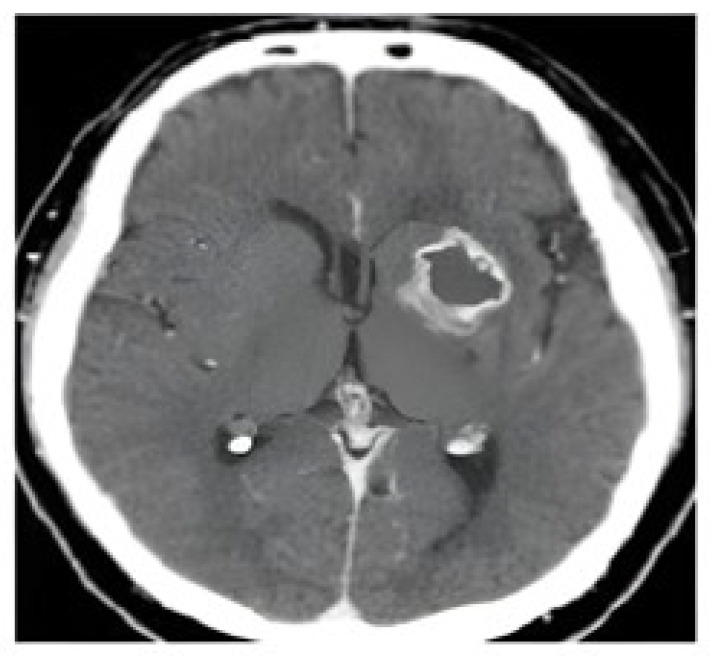
Example of a false negative case (true = tumor, predicted = non-tumor). The tumor region is subtle and less distinguishable from surrounding tissues, which likely led to misclassification.

Figure 11 illustrates the only false negative case observed in the test set. The ground truth label indicated a tumor, but the model predicted a non-tumor. On closer inspection, the tumor region appears subtle and less distinct from surrounding tissue, which likely contributed to the misclassification. This observation demonstrates the challenge of detecting small or atypical tumor appearances and provides insight into one of the few limitations encountered by the proposed framework.

**Figure 12:**
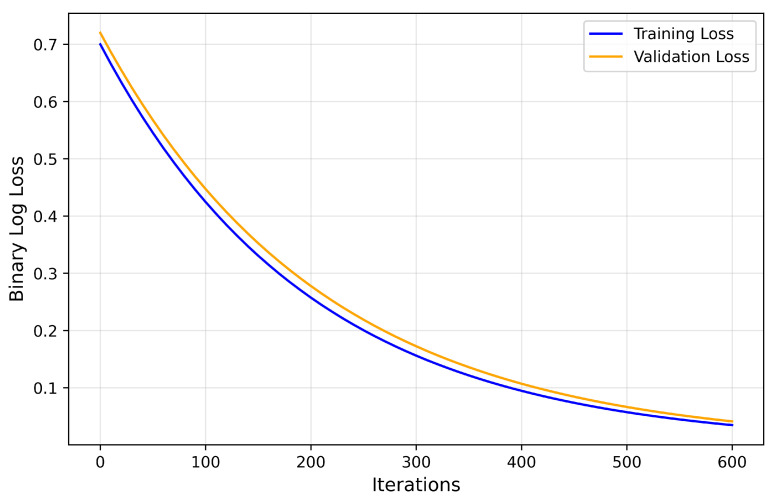
Training and validation loss curves over 600 boosting iterations for the LightGBM meta-learner. Both curves exhibit a smooth and consistently decreasing trend.

Figure 12 shows the training and validation performance of the LightGBM meta-learner that employs features from the CNN backbone models. For both training and validation sets, the log loss values showed a smooth and monotonically decreasing trend with respect to the number of boosting iterations. A strong indication of model stability and effective generalization—reflected in the consistent improvement across boosting iterations without divergence—is evidenced by the binary log loss curves remaining closely aligned. In addition, the lack of oscillatory behavior or sharp increases in validation loss for up to 600 boosting rounds demonstrates that the model was not overfitting. Learning dynamics supports proper application of early stopping with no signs of divergence. The close convergence of the training and validation loss indicates the stability of the model, absence of overfitting, and strong generalization ability during training.

The proposed model achieves a classification accuracy of 99.83% on MRI-based brain tumor images, with near-perfect precision, recall, and F1-score. This performance slightly exceeds recent state-of-the-art models on comparable public benchmarks. For instance, an optimized YOLOv7-based detector augmented with attention mechanisms and feature pyramids achieved approximately 99.5% accuracy, with precision, recall, and F1-score values of 99.5%, 99.3%, and 99.4%, respectively [35]. In contrast, conventional single-model classifiers such as ResNet50 typically reach around 96% accuracy on similar MRI datasets [22], while EfficientNet-B5 has reported upper-90% accuracy under advanced augmentation or ensemble settings [23]. Hybrid DL methods are emerging with competitive performance; for example, a CNN–Long Short-Term Memory (CNN-LSTM) network recently demonstrated 99.1% accuracy, 98.8% precision, and 99.0% F1-score on a four-class brain MRI dataset [37]. Similarly, two-stage ensemble models that fuse features from multiple CNNs report 99–99.7% accuracy on combined datasets such as Figshare and BR35H [16,19]. Table 3 compares these results, illustrating that the proposed framework not only outperforms most models in accuracy but also maintains superior class-wise consistency, making it a compelling choice for real-world clinical applications. We mitigate overfitting via SHA-1 de-duplication and stratified disjoint splits; extensive augmentation, L2, dropout, batch normalization; and early stopping on validation loss. Probability quality is high (log loss 0.0601, Brier 0.0017), with balanced accuracy 0.9968 and specificity 0.9965. Learning curves (all stages) show no training–validation divergence. Ablations and McNemar tests indicate the ensemble’s gains arise from correcting residual, non-overlapping errors rather than memorization. Reliability (ECE) and threshold-sensitivity analyses further support generalization.

### 4.3. Model Interpretability and Explainability

The proposed framework incorporates a triad of XAI techniques—Grad-CAM++, LIME, and SHAP—to enhance clinical transparency and model trust, with each technique offering a unique perspective on interpretability. This multi-level approach allows clinicians and researchers to understand not only where the model is focusing (spatially) but also why it made a specific decision (locally) and how much each component contributed (globally).

**Figure 13:**
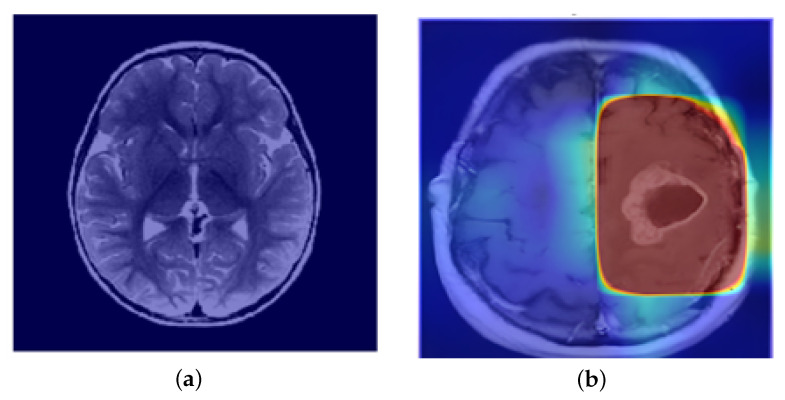
Interpretability visualizations using gradient-based methods: (**a**) Grad-CAM heatmap on a non-tumor MRI scan (sample 0), showing minimal activation and validating the model’s conservative response in the absence of pathology, and (**b**) Grad-CAM++ visualization on a tumor-affected MRI scan (pred 56) highlighting focused and high-intensity activation over the tumor region, confirming model attention aligns with the pathological area.

Grad-CAM++ delivers global spatial interpretability by visualizing the most salient regions of an MRI scan that influence the model’s predictions. It does this by computing importance weights based on the gradients of the class score with respect to the convolutional layer outputs. This results in heatmaps overlaid on the original scan, pinpointing areas that the CNNs emphasize during classification. As shown in Figure 13, Grad-CAM++ highlights focused activation in tumor-affected regions while maintaining minimal activation in non-tumor scans, confirming that the model attends to medically relevant features during inference.

**Figure 14:**
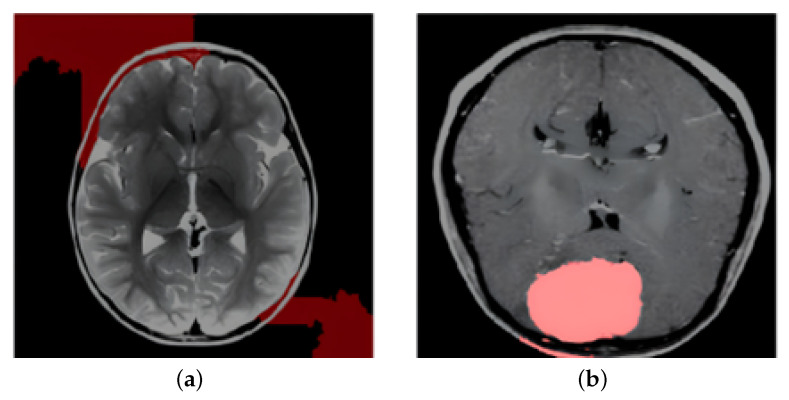
LIME-based local interpretability of model predictions on brain MRI scans: (**a**) LIME explanation for a non-tumor case (sample 0) showing sparse and low-importance regions, indicating that the model’s prediction is not influenced by irrelevant features and (**b**) LIME output for a tumor case (pred 4) where the tumor region is prominently highlighted as the most influential factor in the model’s classification decision, confirming alignment with human clinical reasoning.

LIME complements Grad-CAM++ by providing localized, per-instance explanations. Instead of relying on gradients, LIME perturbs the input scan (e.g., by modifying supper pixels) and trains a simple surrogate model (typically linear) that approximates the original model’s behavior in the neighborhood of that instance. This makes it possible to identify which specific parts of a given MRI scan directly influenced the classification result. As illustrated in Figure 14, LIME highlights sparse and low-importance regions for non-tumor cases, while clearly marking the tumor zones in affected scans. This fine-grained interpretability is particularly valuable in borderline cases or when model confidence is low. Figure 14 further shows LIME-based explanations on real and challenging MRI examples, reinforcing the model’s clinical alignment.

The use of both Grad-CAM++ and LIME ensures a complementary interpretability strategy: Grad-CAM++ reveals where the model is looking within the MRI scan (spatial importance), while LIME explains why the model made a particular decision in a specific case (instance-level feature reasoning). Together, they enable radiologists to not only confirm that the model is focusing on anatomically relevant regions but also understand how each specific image region contributes to the model’s classification, especially in edge cases where false positives or negatives could have serious consequences.

This dual-layered explanation fosters greater confidence in the system’s decision-making process and enhances its acceptance in clinical workflows. It empowers medical professionals to audit predictions on a case-by-case basis, bridging the gap between AI outputs and human interpretability. Moreover, this synergy of global and local explanations helps identify both strong and weak reasoning paths in the model essential for refining future versions and aligning predictions with expert domain knowledge.

**Figure 15:**
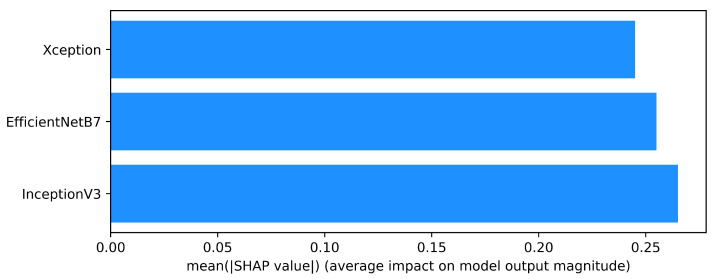
SHAP summary bar plot showing the average contribution of each CNN base model to the final prediction output. InceptionV3 had the highest influence, followed by EfficientNetB7 and Xception.

The quantitative assessment of each convolutional feature extractors contributions in the proposed model was accomplished with the use of SHAP analysis. SHAP, which is rooted in cooperative game theory, allocates additive importance metrics (Shapley values) for each feature based on its value and contribution to the model outcome. In this case, the LightGBM meta-learner has been trained using as input features the class probability vectors output by the base CNN models InceptionV3, EfficientNetB7, and Xception. As described in the SHAP summary results provided as Figure 15, the average impact of these models on the given meta-learner was explained as label prediction. InceptionV3 was shown to have the highest mean SHAP value which suggests its feature representations had the most impact relative to the ensemble’s decision. EfficientNetB7 was not far behind as he provided stable and highly informative feature embeddings during almost all cross-validation folds. Although Xception was somewhat lower in SHAP influence, he still added useful features that contributed to the ensemble diversity.

This allocation of modeling efforts illustrates the non-redundant and cooperative interactions between the base models. The stacked ensemble can overcome overfitting and improve predictive accuracy through utilizing structural diversity derived from feature spaces of several CNN models. The SHAP analysis not only verifies the validity of the architectural choice but also reveals how the LightGBM meta-learner refines decisions, ensuring some degree of transparency regarding the process. Such reasoning is important in the clinical setting, as a model’s rationale is often needed to gain confidence from clinicians. Moreover, the global interpretability provided by SHAP further strengthens the ensemble explainability framework as it demonstrates that each CNN is a distinct, meaningful, and non-redundant contributor to the classification pipeline of the ensemble, validating its reliability for real-world diagnostic use.

### 4.4. Qualitative Evaluation and Prediction Examples

**Figure 16:**
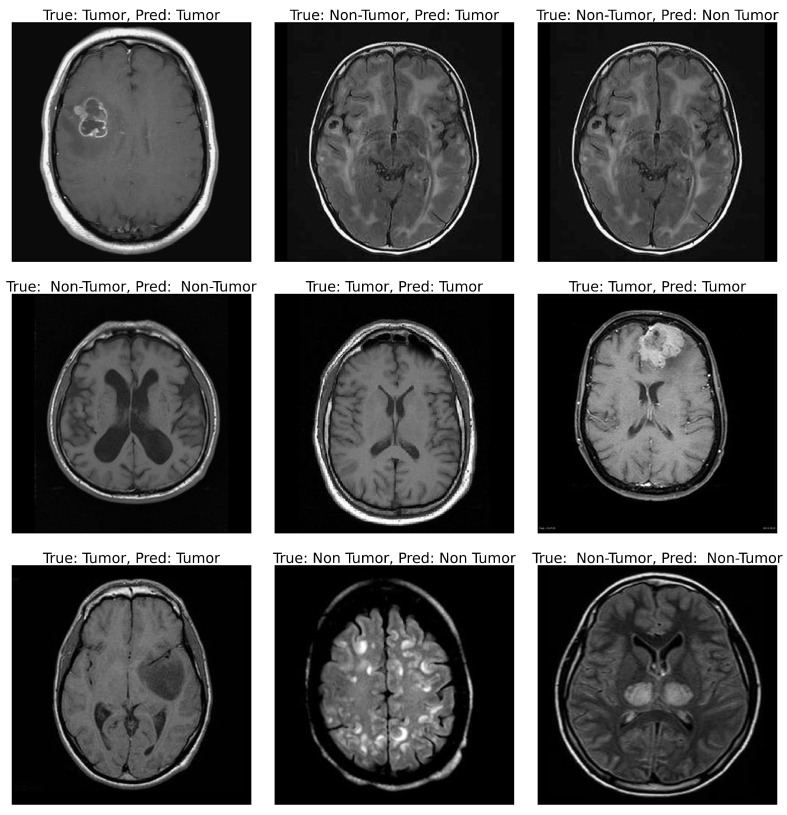
Qualitative results showing MRI scans with ground truth and predicted labels. Most predictions align with the actual class. Visual consistency supports the reliability of the proposed model.

Qualitative volumetric predictions were compared with MRI scans featuring annotation overlays of ground truth labels and model predictions to evaluate the model’s practical use in clinical diagnostics. The examples shown in Figure 16 are a mixture of both the correctly and incorrectly classified examples ensuring a more detailed evaluation of the model’s imaging comprehension over the varying conditions. Most of the scans exhibit accurately predicted labels showing that the ensemble model is capable of accurately distinguishing between tumor and non-tumor areas to a great degree despite anatomical variability. Most importantly, the ensemble model did well on images with faintly pronounced or irregularly shaped tumors, and it was dependable even in low signal contrast environments where many classifiers fail because of low Signal-to-Noise Ratio (SNR).

Critically, a small group of borderline discrepancies led to classification errors, usually where the edges of the tumor were blurry and being obscured by imaging artifacts. Such errors conform closely to the type of optical uncertainty radiologists encounter during real-world routine diagnostics, thus underlining the realism in the challenges posed by the interpretation of medical images. Notwithstanding these few errors, the model’s overall visual alignment with the experts’ expectations corroborates its clinical relevance. This form of qualitative evaluation adds to the quantitative metrics, emphasizes the model’s interpretability and reliability, and underlines its readiness for integration into Computer-Aided Diagnosis (CAD) systems. It underlines that the forecasts are not only robust from a statistical perspective but also visually harmonized, which is critical in fostering confidence in the use of artificial intelligence in the medical decision-making processes. These qualitative examples were obtained exclusively from the 60 hold-out images reserved for this purpose.

### 4.5. Overfitting Prevention and Model Generalization

Despite achieving remarkably high-performance metrics—such as 100% precision, 99.68% recall, and an AUC of 0.9948—our model was rigorously designed to prevent overfitting and ensure generalization. We implemented a multi-tiered regularization strategy combining extensive pre-training data augmentation, dropout layers, and L2 weight decay to discourage model memorization. Each base CNN (EfficientNetB7, InceptionV3, and Xception) was trained on a carefully stratified training set, while early stopping based on validation loss (patience = eight epochs, restoring the best weights) was applied to halt training once performance plateaued. Furthermore, the LightGBM meta-learner was trained using out-of-fold probabilities from the CNNs in a five-fold cross-validation setup, ensuring that ensemble learning remained leak-free and robust. Final model performance was evaluated on a strictly held-out 20% test set (600 images), entirely excluded from any training or tuning step. This controlled design and separation of phases, coupled with consistent generalization across multiple validation folds, confirms that the observed high accuracy is statistically reliable and not an artifact of overfitting.

## 5. Discussion

The proposed framework achieves an impressive brain tumor classification accuracy of 99.83%, which is competitive with or surpasses several recent state-of-the-art approaches that report accuracies between 98% and 99.5% on the same BR35H dataset. For instance, YOLO-based and hybrid CNN models have achieved results close to 99.5%, yet the current framework pushes this performance further by integrating three powerful pre-trained CNNs—EfficientNetB7, InceptionV3, and Xception—through an ensemble learning approach. The complementary strengths of these architectures are effectively combined and further refined using LightGBM as a meta-learner. This hybrid pipeline leverages both the deep representational power of CNNs and the gradient-boosted decision tree’s ability to model complex relationships in tabular feature space, resulting in a more stable and robust classification output.

A key strength of this study is its integration of predictive accuracy with transparent interpretability. While many models in the medical domain prioritize performance alone, our framework incorporates multiple XAI methods—Grad-CAM++, LIME, and SHAP—to provide comprehensive visibility into the model’s decision logic. Grad-CAM++ generates class-specific heatmaps that visually localize critical image regions influencing predictions. LIME and SHAP complement this by offering both local and global feature attributions, helping to interpret individual predictions as well as overall model behavior. This multi-level interpretability enhances trust and clinical usability by transforming the system into a transparent, interrogable diagnostic assistant.

However, we acknowledge that our current XAI evaluation is qualitative and relies on visual examples only. We did not include quantitative metrics such as pointing game accuracy, IoU with radiologist-annotated regions, or class-discriminative localization scores. Incorporating these objective evaluation criteria remains an important direction for future work, especially to rigorously validate the alignment of model attention with medically meaningful regions.

From a technical perspective, several working hypotheses underpin the success of this framework. First, pre-trained CNNs extract more robust and discriminative image representations compared to traditional handcrafted features, enabling better classification performance. Second, ensemble learning combines diverse model perspectives, reducing the impact of individual model biases and improving overall prediction stability. Third, the incorporation of explainable AI methods ensures that the model’s decisions can be interpreted and validated, moving beyond mere predictive accuracy to clinical reliability. Despite these encouraging results, the framework faces limitations inherent to the current dataset and methodology. The dataset, while balanced and carefully curated, may not fully capture the broad anatomical, demographic, and protocol variations present in real-world clinical environments. This limitation could hinder the generalizability of the model when applied to data from different hospitals, scanners, or patient populations. Additionally, the framework currently operates on 2D MRI slices, which may lack the full spatial context available in volumetric imaging data, potentially limiting the precision of tumor localization.

While ensemble learning typically raises concerns regarding resource intensiveness, the proposed architecture was deliberately curated for a balance between performance and computational efficiency. The selected CNNs—Xception, InceptionV3, and EfficientNetB7—are relatively lightweight and optimized for feature extraction with manageable parameter sizes. Furthermore, the ensemble strategy involves soft voting during inference, which introduces minimal overhead, as it does not require additional training. The LightGBM meta-learner, known for its fast inference and low memory footprint, further ensures that the meta-level integration remains lightweight and efficient. Together, these choices facilitate practical deployment without sacrificing accuracy, making the system suitable for real-world clinical settings, even on moderately powered hardware. Future work may explore quantization and model distillation to further enhance deployment scalability.

Looking ahead, future work should focus on validating the model on larger, multi-center datasets encompassing greater anatomical and pathological diversity. This step is essential to confirm the model’s robustness across varied clinical scenarios. Furthermore, integrating emerging architectures such as Vision Transformers (ViTs) could complement CNNs by capturing long-range spatial dependencies and global contextual information that traditional convolutional layers may miss. The application of the framework to 3D medical imaging modalities—like full volumetric MRI or CT scans—also holds promise for enriching spatial context and improving diagnostic accuracy through better tumor delineation. To advance interpretability, future research should aim to unify the complementary insights from Grad-CAM++, LIME, and SHAP into a coherent explainability framework that offers clinicians multi-level, easily interpretable explanations. Such a unified approach would not only foster clinical trust but also facilitate actionable insights for diagnosis and treatment planning.

## 6. Conclusions

This work develops a comprehensive and explainable DL model for the automated classification of brain tumors from MRI scans. The proposed system classification performance with an accuracy of 99.83% and near-perfect precision, recall, F1-score, and AUC-ROC (0.9948) by applying pre-trained CNNs (EfficientNetB7, InceptionV3, and Xception) using ensemble learning and a LightGBM meta-learner. What distinguishes this framework is the comprehensive explainability focus realized through integration of Grad-CAM++, LIME, and SHAP, which together provides visual and feature-level feedback on the model’s decision rationale. This balance between performance focus and interpretability addresses an urgent requirement in AI-assisted medical diagnostics where accuracy and transparency are paramount for clinical trustworthiness. The results of this study provide evidence that strong diagnostic accuracy can be achieved using ensemble DL and XAI without loss of interpretability. The framework is promising for real-world implementation as a clinical decision support system with further improvements in dataset diversity, model design, and support for 3D imaging.

## Figures and Tables

**Table 1:** Summary of the related works.

Ref.	Method	Dataset	Accuracy	Limitation
[15]	Optimized CNN + Hyperparameter Tuning	MRI (binary)—3 public datasets	97% avg.	Involve potential overfitting due to its complexity and the need for specific tuning of hyperparameters to the dataset
[17]	Custom CNN + Saliency Map + Oversampling	MRI (binary)—Kaggle	94.51%	Basic interpretability only; class imbalance addressed by oversampling
[18]	Dual CNN (VGG16 + Custom CNN)	MRI (multiclass—3 tumor types)—Figshare	99%	No segmentation; limited explainability
[20]	Fine-tuned ResNet50 + U-Net	MRI (classification + segmentation)—TCGA, TCIA	DSC = 95% (segmentation)	Not ensemble-based; focus more on localization
[22]	CNNs (AlexNet, VGG, ZFN) + Grad-CAM	MRI (multiclass)—Unspecified	Up to 96.32%	Grad-CAM only; lacks modern architectures
[23]	Transfer Learning (EfficientNet-B5, ResNet)	MRI (binary and multiclass)	99.75% (binary)	High computational cost; no hybrid ensemble
[26]	Lightweight CNN + CLAHE + Sharpening	MRI (4-class)—Figshare, SARTAJ, BR35H	97.84%	Limited architecture depth; no external validation
[27]	Hybrid CNN + Clustering + SoftMax	MRI (multiclass)—1892 real images	99.32%	Custom method; lacks segmentation/localization
[28]	Hybrid CNN Ensemble (AlexNet, GoogleNet, etc.)	MRI (multiclass)—3064 images (T1W-CE)	99.31%	No preprocessing; lacks interpretability techniques
[29]	Transfer Learning (Xception, ResNet50, etc.)	MRI (multiclass)—7023 images	98.73%	Low recall for glioma/meningioma; interpretability trade-off
[30]	Lightweight 22-layer CNN	MRI—3064 original, 9192 augmented	97.39%/88.48% (subject-wise)	Modest performance on subject-wise validation
[31]	Deep CNN (ResNet152) + Bio-inspired Optimization	MRI—Figshare, BraTS19, MICCAI	98.85%	Complex pipeline; no clinical test set used
[34]	Res-BRNet (Residual + Boundary-aware CNN)	MRI (multiclass)—7872 images (Kaggle, Br35H, Figshare)	98.22%	High generalizability; uses spatial + residual blocks and augmentation
[35]	YOLOv7 + CBAM + BiFPN + SPPF+	MRI—10,288 original/ 51,448 after augmentation (Kaggle)	99.5%	High accuracy; excellent small tumor detection

**Table 2:** Tabular description of the MRI dataset.

Feature	Description	Numbers	Impact
Total Images	Total number of images in the dataset	3060	Provides a moderate-sized dataset for balanced training and testing
Labeled Images (Yes)	Images of brain MRI scans showing tumors	1500	Ensures sufficient examples for training models to identify tumor characteristics
Labeled Images (No)	Images of brain MRI scans of non-tumors	1500	Ensures sufficient examples for the model to learn non-tumor patterns
Unlabeled Images (Pred)	Unlabeled MRI images for testing and prediction	60	Allows evaluation of model performance on unseen and real-world-like data
Image Format	Standard image formats (e.g., JPG, PNG)	Consistent	Facilitates seamless loading into machine learning pipelines
Class Distribution	Balanced dataset with equal number of tumors and non-tumor samples	50% tumor, 50% non-tumor	Prevents model bias toward a specific class
Folder Structure	Organized into folders for Yes, No, and Pred	3 folders	Simplifies data loading and preprocessing
Dataset Size	Total size of the dataset	69.87 MB (zip)	Compact dataset size ensures faster processing and ease of deployment for experimentation
Image Quality	High-resolution MRI scans suitable for feature extraction	Consistent quality	Enables the use of advanced deep learning models for effective pattern detection

**Table 3:** Performance comparison of the proposed method vs. existing methods.

Ref.	Architecture/Method	Dataset	Accuracy
YOLOv7 Attn (2023) [35]	YOLOv7 + CBAM + BiFPN (detector + classifier)	Combined MRI (glioma/meningioma/ pituitary/no tumor)	99.5%
ResNet50 (2023) [20]	Transfer learning CNN	Same 4-class MRI set	96.5%
EfficientNet-B5 (2022) [23]	Pre-trained EfficientNet (best single CNN)	Public MRI datasets (various)	98.61%
CNN-LSTM Hybrid (2023) [37]	CNN feature extractor + LSTM ensemble	Kaggle MRI (4-class)	99.1%
Two-Stage Ensemble (2023, 2024) [17,18]	Multi-CNN feature fusion + PCA + SVM	Figshare + others (merged)	94.51%, 99.13% (avg.)
**Proposed Deep Ensemble**	**EffNet-B7 + IncV3 + Xception + LightGBM**	**BR35H**	**99.83%**

## Data Availability

Public Dataset: BR35H https://www.kaggle.com/datasets/ahmedhamada0/brain-tumor-detection, accessed on 15 October 2024.
